# Energy Efficient Medium Access Control Protocol for Clustered Wireless Sensor Networks with Adaptive Cross-Layer Scheduling

**DOI:** 10.3390/s150924026

**Published:** 2015-09-18

**Authors:** Maria Sefuba, Tom Walingo, Fambirai Takawira

**Affiliations:** 1 Discipline of Electrical, Electronic and Computer Engineering, University of KwaZulu Natal, Durban 4041, South Africa; E-Mail: walingo@ukzn.ac.za; 2 School of Electrical and Information Engineering, University of Witwatersrand, Johannesburg 2000, South Africa; E-Mail: fambirai.takawira@wits.ac.za

**Keywords:** medium access control (MAC), scheduling, diversity, channel quality indicator, intra-cluster, inter-cluster

## Abstract

This paper presents an Energy Efficient Medium Access Control (MAC) protocol for clustered wireless sensor networks that aims to improve energy efficiency and delay performance. The proposed protocol employs an adaptive cross-layer intra-cluster scheduling and an inter-cluster relay selection diversity. The scheduling is based on available data packets and remaining energy level of the source node (SN). This helps to minimize idle listening on nodes without data to transmit as well as reducing control packet overhead. The relay selection diversity is carried out between clusters, by the cluster head (CH), and the base station (BS). The diversity helps to improve network reliability and prolong the network lifetime. Relay selection is determined based on the communication distance, the remaining energy and the channel quality indicator (CQI) for the relay cluster head (RCH). An analytical framework for energy consumption and transmission delay for the proposed MAC protocol is presented in this work. The performance of the proposed MAC protocol is evaluated based on transmission delay, energy consumption, and network lifetime. The results obtained indicate that the proposed MAC protocol provides improved performance than traditional cluster based MAC protocols.

## 1. Introduction

The self-organizing nature of micro sensors in Wireless Sensor Networks (WSNs) and their ability to operate without support of predetermined infrastructure makes them effective for data gathering in a variety of areas, even in harsh environments. Energy consumption however remains one of the main design challenges in WSNs, due to the limited energy resource that is supplied by the batteries in the sensor node. It is usually unfeasible to recharge or replace the batteries once the sensor nodes have been deployed due to inaccessible terrains and enormous deployment scale [1]. A thoughtful design of WSNs is required to provide significant benefit to network lifetime by being energy efficient. Research has shown that the sensor node utility that drains the most energy is the radio module during communication mode [2]. The causes of energy waste in the radio module of the sensor node have been identified mainly as idle listening, collisions, overhead and overhearing.

MAC protocols are employed to arbitrate access to the shared medium to avoid different causes of energy waste, and at the same time to efficiently share the medium resources among multiple sensor nodes [3]. Energy efficient MAC protocols control the duty cycle of the sensor nodes, based on the availability of traffic, minimizing idle listening leading to reduced energy waste [1,4]. MAC protocols use efficient schedulers to adapt to different traffic patterns of the network. Most schedulers are based on the sensor node traffic without considering the energy remaining in the nodes. The use of the remaining energy of sensor nodes in determining sensor nodes’ schedule is important in improving the network energy performance. The large scale deployment of sensor nodes also contributes to the high communication packets overhead in WSNs, as all nodes report their sensed data to the base station, which results in a lot of energy waste. To alleviate this problem, clustering has been widely adopted in the design of WSNs [5]. Clustering reduces the number of transmissions to the base station as the CH is responsible for the communication of each cluster. Clustering is known for its scalability as it provides load balancing and efficient resource utilization by grouping the nodes within a geographical neighborhood into a cluster.

WSNs utilize the wireless channel for their communication, and therefore are prone to fading due to the unreliable nature of the wireless channel. This leads to packet errors, which requires packet retransmission, resulting in energy waste. Currently researchers employ cooperative diversity to mitigate the effects of fading in WSNs, by using relay nodes to cooperate in the communication [6]. The enhanced reliability through the use of cooperating terminals reduces the need for retransmission which effectively leads to energy conservation. Cooperative diversity utilizes relay selection to choose the best relay nodes based on the channel quality of the relay link, and seldom based on the combination of distance and residual energy of the relay nodes which is also a vital instrument for the efficiency of WSNs.

The aim of this work is to design an energy efficient MAC protocol for cluster-based wireless sensor networks, based on distance, residual energy, and channel quality, to improve the energy and delay performance of WSNs. The remainder of this paper is organized as follows: related work is discussed in Section 2, followed by the system model and detailed description of proposed MAC protocol in Section 3. Section 4 presents description of the analytical channel and energy model for proposed protocol. The simulation based performance and analytical performance evaluation is discussed in Section 5, and lastly the conclusions are presented in Section 6.

## 2. Related Work

LEACH [2] and LMAC [7] are built on the concept of TDMA and allocate single independent transmission slots to each sensor node in a cluster. The TDMA-based schedule provides collision free slot allocations to sensor nodes, leading to reduced energy waste; however it is faced with a challenge of idle listening on nodes that do not have data to send during their allocated slots. ETDMA [8] extends the operation of TDMA to further reduce energy consumption due to idle listening, by turning off the radio modules of the sensor nodes that have no data to send during their assigned slots. Though energy is conserved by avoiding idle listening, the protocol incurs a lot of delay due to the allocated unutilized slots. BMA [9] is an intra-cluster communication protocol for large-scale cluster-based WSNs intended for event-driven applications. It reduces energy waste by avoiding idle listening by scheduling only nodes that have data to transmit. The protocol allocates a single transmission slot to each node that has data to transmit. The enhancement of the protocol, BMA-RR [10] provides variable data slots to sensor depending on their load. The scheduler is based only on the available packets on the sensor node, without considering the remnant energy of the sensor nodes, which could enhance the schedule performance. The data transmission of this protocol also does not feature cooperative transmission to address wireless channel reliability issues in WSNs.

The cooperative communication research presented in [11] introduces both proactive and reactive protocols of cooperative communication; the protocols are both efficient, but due to the vast deployment of sensor nodes, there is high possibility of overhead incurred by the system due to the receipt of control signals from individual nodes by neighbors. Work done in [12] presents a threshold-based adaptive relay selection scheme, which uses a threshold to select the relays for cooperation. The drawback of this model is its use of multiple relays while sometimes only one relay might have enough channel gain to make a successful transmission, leading to unnecessary energy consumption due to transmission of multiple relay terminals. CPS-MAC [13] selects the relay nodes based on the buffer state and the channel state information (CSI) obtained through the exchange of control messages. The protocol however does not consider the remaining energy of the relay nodes, which can cause early network partitioning if relay nodes run out of energy. A close relation to our cooperative model is WcoopMAC [14], a cooperative MAC protocol adopting decode and forward technique for relaying of data to improve the energy performance of WSNs. The protocol selects the relay nodes based on the residual energy information and CSI, however it does not consider the relay node distance. The protocol is reactive, making an inefficient use of the channel as the direct link is always used regardless of the channel conditions, which leads excessive energy waste due to retransmissions.

Although the effects of the techniques mentioned in this work and applied to the communication protocols have been independently investigated, not many protocols have applied cooperative diversity in clustered WSNs. This work makes the following contributions: first a cooperative MAC protocol featuring CQI, energy and distance for relay selection combined with cross-layer based scheduling is presented. Secondly, an analytical model for the energy consumption and delay for the proposed protocol is developed. This work differs from other works as it incorporates the effects of clustering and cooperative diversity in a scheduled MAC for WSNs, and does not examine the two techniques independently. This work also proposes a new scheduling algorithm at cluster level with the aim to improve energy performance among nodes with varying loads within the cluster such that all nodes are given a fair chance of transmission.

## 3. System Model

**Figure 1:**
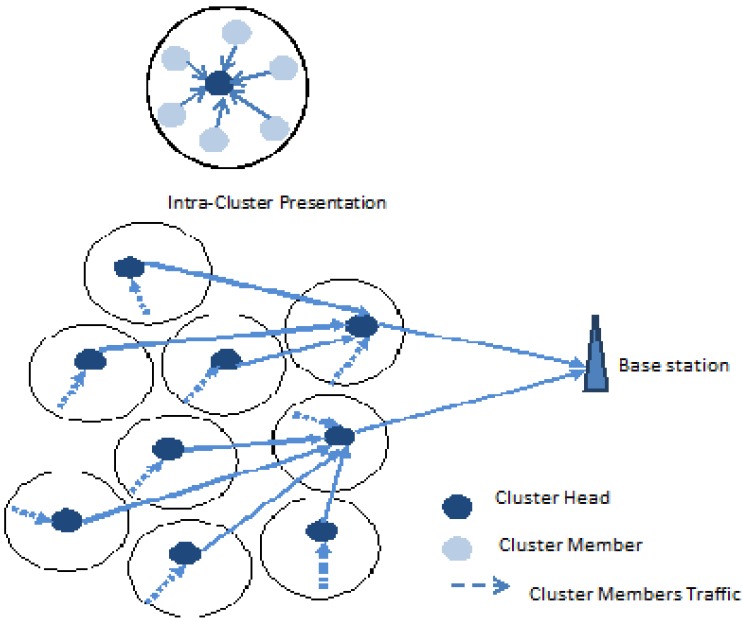
Selection Relaying Cooperative WSN.

The system architecture used in this work is adopted from LEACH [2], that has the basic system model for a cluster-based WSNs scenario. It has proved to have energy savings above a factor of 7 compared to flat architecture. The network is composed of sensor nodes randomly distributed in a geographical area, and grouped into clusters. There are Normal Sensor Nodes (SN) and Cluster Heads (CH). Each SN in every cluster communicates directly with the CH and every communication within the cluster is handled by the CH. A Base Station (BS) with unlimited energy is located far away from the sensor network as illustrated in Figure 1.

A CH in each cluster is responsible for communicating the aggregated data of the cluster to the BS. The model is further enhanced to meet our communication scenario, communication between the CH and BS can either be direct or cooperative. Direct communication is used when the channel condition is good. If the channel conditions are fair, communication can be either direct or cooperative, based on the CQI and distance ratio between the CH and BS. If the channel is bad, cooperative communication is considered. For cooperative communication, a relay cluster head (RCH) is selected to aid the CH communication to BS. RCH selection is based on its distance away from the BS, its remaining energy level and the CQI along its link. The CH communicates with RCH, which then relays the information to the BS.

### 3.1. The Proposed MAC Protocol

**Figure 2:**
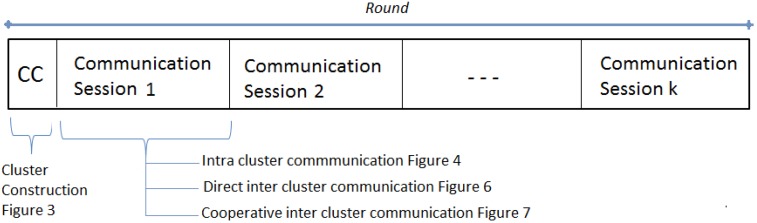
MAC Communication Round.

The proposed MAC protocol is time slotted and its operation is separated into intra-cluster communication and inter-cluster communication. The inter-cluster communication is further divided into communication rounds as illustrated by a complete inter-cluster communication frame in Figure 2. Each round is formed by Cluster Construction (CC) and a number of sessions. The duration of a communication round is fixed and number of sessions is decided on implementation depending on data traffic of the application.

A communication session consists of contention (Cont.) period, a schedule (Sch.) slot, data communication (Dt.) slots and variable sleep slot period. The sleep period varies depending on the traffic available during each session. Each cluster follows its independent intra-cluster communication frame.

### 3.2. Cluster Construction

**Figure 3:**
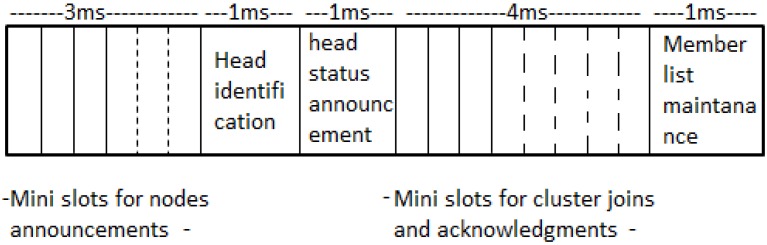
Cluster Construction.

The clusters are formed and renewed during the cluster construction period which occurs at the start of a communication round. During this period, SNs determine their energy with respect to others within its vicinity. The contention time is divided into mini-slots, within which each node announces their energy level and coordinates. Each mini slot is built of a short inter-frame space (SIFS) and a communication slot. A node waits a SIFS time before it transmits to avoid collisions. When all the nodes have sent their announcements, a cluster head is identified based on the remaining energy and the SN with higher energy level elects itself as a CH; then it announces its head status to other SNs in the next slot. The SNs close to the CH decide to join its cluster, using CSMA/CA contention on the allocated mini-slots. CH then forms and maintains a member list table as indicated by cluster formation frame in Figure 3. The cluster construction process is further illustrated by Algorithm 1.

**Algorithm 1** Cluster ConstructionSN announces its energy level and coordinates within its neighborhood,SNs determine their energy level with respect to other nodes within same region,**If**
*SN’s energy is highest*
**then** SN elects self as CH, CH announces head status, CH waits for join request,**Else** SN waits for CH announcements, Determine CH with shortest communication distance,**If**
*CH’s distance shortest*
**then**   SN sends cluster join request to CH,**End if****End if** CH forms members list table,CH announces the cluster members list

### 3.3. Intra-Cluster Communication Session

**Figure 4:**
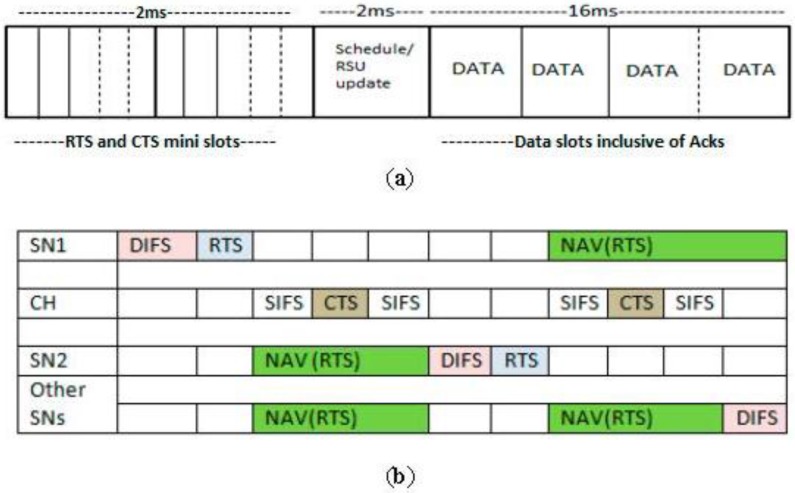
(**a**) Nodes’ Communication Session; (**b**) Nodes’ Contention Window.

After the clusters are formed, communication takes place within each cluster during communication sessions. In each session, the contention time for the handshake mechanism is divided into mini slots within which the nodes indicate their intention to transmit data. The nodes contend for the medium using CSMA/CA employed by IEEE 802.11 MAC protocol, through RTS packet transmission to the CH. During the Schedule period the CH uses the source nodes’ information on the received RTS packets from the SNs, and prepares slot reservations based on an adaptive cross-layer schedule. The cross-layer schedule uses the remaining energy and the amount of data traffic on each SN to decide which nodes should be scheduled first. The nodes with the least energy to packets ratio, calculated from the RTS packet information, are scheduled earliest. If the destination intended for the source packets is not available or dead, the node will not be scheduled but will drop such packets. Once the schedule is done, the CH broadcasts the schedule to all nodes within the cluster. After the schedule announcement, data communication takes place, according to the schedule. Scheduled nodes transmit at their scheduled time, and when a node completes transmission, it goes to sleep until next session. The nodes that are not scheduled sleep until the next session begins. Every node follows a transmission frame, subdivided into time slots as shown in Figure 4a. The time slots are decided based on the amount of communication required for each packet and the bandwidth utilization depending on whether a message is a unicast or a broadcast message. The session communication is outlined in Algorithm 2.

The frame indicates the communication activities of a node from one communication frame to the next. The node sends a packet in one frame and receives in the next frame. Figure 4b illustrates the detailed cluster notes communication during the contention window as indicated by the RTS and CTS slots the in Figure 4a.

**Algorithm 2** Intra-cluster Data Communication SessionCommunication session begins,Nodes check their buffers for packets,**If**
*packets available and node is SN*
**then** Contend using CSMA/CA for medium and send RTS,**Elseif**
*node is CH*
**then** Receive RTSs, Send CTSs,**End if****If**
*medium idle for SIFS +DIFS*
**then**CH prepares nodes’ schedule according to Algorithm 3,**End if**CH Broadcast schedule to all members,SNs identify their transmission slots from the received schedule,Nodes Tx and Rx data in allocated slots,CH receives data from the SNs in allocated slots,CH aggregates data to send to BS.

#### Cross-Layer Scheduling

In WSNs the data traffic load varies on individual nodes, as well as the energy level from one sensor node to another. A node may have lots of data traffic to transmit, and may be prioritized over other nodes. This can present fairness issues for other nodes which still need to transmit their data, and some of which may have insufficient resources. We present a cross-layer scheduling algorithm adaptive to the data traffic and available energy resources of the sensor node. The schedule stores the data requests of sensor nodes in a job array, and the energy levels of all source nodes in the corresponding fields of the resources array.

The schedule defines a medium access decision parameter $\varphi_{i}$ for each of the nodes, which is the ratio of the number of packets available to the remaining energy level of a node. The schedule checks the slot schedule array to find if there are available slots, if there are still available slots the schedule checks the $\varphi_{i}$ values of each node and priority of access is granted to a node that has the highest value. The node is then allocated the particular slot. The schedule then reduces the job array for the node by 1 and updates the energy level. If the packet to energy ratio is same for some nodes, the schedule will prioritize the node that was scheduled in previous slot. The process is repeated while there are still available slots to schedule and there are jobs in the job array as illustrated by Algorithm 3.

**Algorithm 3** Scheduling AlgorithmCH checks available slots,**While**
*available_slots > 0*
**do**  Store the transmission request in job array,  Store the energy level in resources array,  CH calculates $\varphi_{i}$ for SNs based on packets and energy level,  Determine SNs with maximum $\varphi_{i}$,  **If**
*SN’s*
$\varphi_{i}$
*= maximum*
**then**    Schedule SN for next available slots for its data transmission,  **Else if**
*SNs have same value of*
$\;\varphi_{i}$
**then**    Check SN scheduled in the previous slot and schedule it for the next available slot,  **End if**  update SN energy level,  update SN packets,  update available slots,**End while**

### 3.4. Cooperative Inter-Cluster Communication

**Figure 5:**
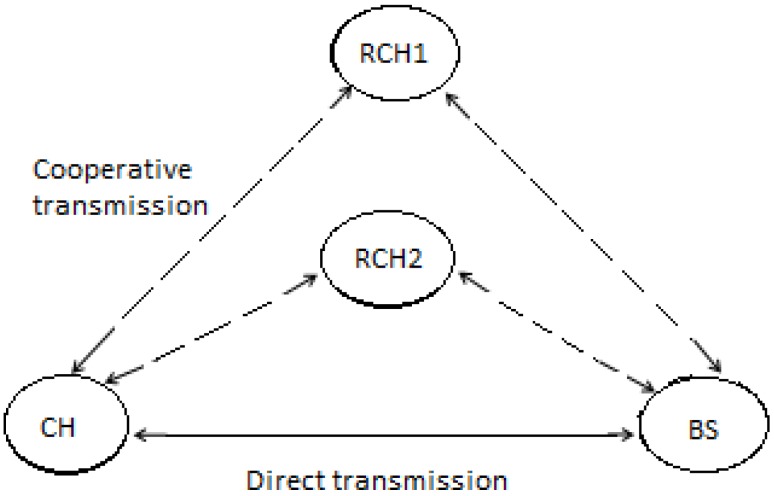
Cooperative Communication.

The inter-cluster communication adopts the basic model for cooperative communication implemented in [15], with an extension of using more than one relay node in this work. The communication involves proactive cooperative communication between cluster heads (CH), the base station (BS) and possible relay cluster heads (RCH) and is illustrated by Figure 5. The relay selection cost is the function of CQI, remaining energy level and the distance away from the BS. The communication is in two phases, direct and cooperative transmission phase.

#### 3.4.1. Direct Transmission

**Figure 6:**
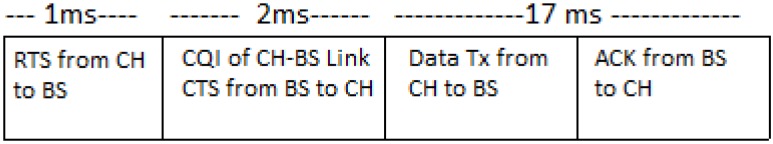
Cluster head direct transmission session.

A CH that has data to send to the BS indicates its request for transmission by sending an RTS packet to the BS. The BS calculates the CQI and attaches its value on the CTS packet to the CH. CH uses CQI and distance to decide the transmission phase. If CQI indicates that the channel is good direct transmission takes place, if the channel is fair and the communication distance is below the separation distance threshold also direct transmission is implemented. A CH then transmits its data packets to the BS. The base station responds with an ACK packet if all data packets are received correctly as illustrated by direct transmission frame in Figure 6, else a NACK is sent, and the CH retransmits the incorrectly received packet to the BS. The direct transmission process is outlined in Algorithm 4.

**Algorithm 4** Direct TransmissionCHs check buffers for packets**If**
*packets available*
**then**Send RTS to BSBS calculates CQI from CH,BS sends CTS to CHCH determines channel conditions based on CQIs, **If**
*(CH to BS channel condition is good) or (fair and distance is less than threshold)*
**then**   CH selects CH to BS channel for transmission,   CH transmit data to BS,   BS sends an ACK to the CH and communication ends, **Else**   Execute **Algorithm 5** **End if****Else**  Continue with intra-cluster communication**End if**

#### 3.4.2. Cooperative Transmission

**Figure 7:**
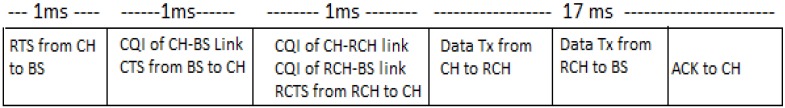
Cluster head cooperative transmission session.

When CH sends RTS to the BS to show their request to send data, the RCHs hear the RTS packet; they calculate CQI for the link between relays and CH. Also, when the BS sends the CTS to CH, the RCHs hear the CTS, they calculate the RCH-BS link CQI which they send to the CH through a RCTS. The CH uses this CTS and RCTS information and distances to decide which transmission phase to follow. If the channel condition is bad between CH and BS or if channel is fair but the distance between CH and BS is huge, an RCH is selected to relay the data. The transmission distance has an effect on the energy consumption by first order radio model [2]. The CH then transmits a relay selection update (RSU) packet to indicate the relay node selected, the other RCH then ignores data transmission from the CH. The CH then sends data to the selected RCH; the RCH then relays the data using facilitation technique to the BS. The RCH then keeps the data until an ACK is received from the BS as illustrated in Figure 7 and outlined in Algorithm 5. If a NACK is received, Selective Repeat Automatic Repeat Request error control [16] is used to retransmit the packets.

**Algorithm 5** Cooperative TransmissionCHs calculate relay selection cost,Determine CH with minimum cost,**If**
*CH’s cost=minimum*
**then**  elect as RCH node,**Else**  Remain as CH,**End if**CH check buffer for packets**If**
*packets available*
**then**  Send RTS to BS and RCHs hear it,  BS calculates CQI from CH,  RCHs calculate CQI for CH to RCH link,  BS sends CTS to CH and broadcast it to RCHs,  RCHs calculate CQI for RCH to BS link,  RCHs send RCTS to CH,  CH determines channel conditions based on CQIs,  **If** (*CH to BS channel condition is fair and distance is greater than threshold) or (CH to BS channel condition is bad)*
**then**    Select RCH with highest CQI,    CH sends a relay selection update to possible relays,    Non selected RCHs retire from communication,    CH transmits data to selected RCH,    RCH relays data to BS using facilitation technique,    BS sends ACK to CH and RCH hears it and communication ends,  **End if****Else**  Continue with intra-cluster communication**End if**

## 4. Analytical Model

The symbols and acronyms for the parameters used in the analysis of the proposed protocol are defined in Table 1.

**Table 1:** Analysis Symbols Table.

Symbol	Definition
*N*	Number of nodes in the network
*CN*	Number of nodes in a cluster
*π_j_*	Network steady state probability
φ	Media access Decision parameter
*L_b_*	Length of packet in bits
$L_{pac_{type}}$	Length of packet(depending on type)
$E_{u}$	Unusable remaining energy
$Pw_{s_{1}}$	Unit energy in Sleep state
$Pw_{s_{3}}$	Unit energy in Back-off state
$Pw_{rx}$	Unit energy in Active receive
$Pw_{tx}$	Unit energy in Active transmit
$E_{s_{i}}$	Energy used in each state
$P_{out}$	Outage probability
γ	Signal to noise ratio
Λ	Arrival rate
$P_{p}$	Packet error rate
$P_{e}$	bit error rate
C	The Channel Capacity
$\rho_{s_{2}}$	Probability of Active State
$\rho_{s_{3}}$	Probability of Back-off State
$\rho_{s_{1}}$	Probability of Sleep State
R	The bit rate in bits/s
$E_{0}$	Initial deployment energy of node

### 4.1. CH Relay Selecion

The relay selection in this communication framework is based on the channel quality of the wireless transmission channel. The statistical time varying nature of the wireless channel between communicating node pairs is modeled as a Rayleigh fading process. The received instantaneous signal to noise ratio is a random variable that is exponentially distributed and has a probability distribution function given by Equation (1) [17]:

(1) f(γ)=1γ¯exp(−γγ¯) γ≥0 

where $\bar{\gamma }$ is the average SNR of the received signal and γ is the SNR threshold. The channel quality indicator is estimated from the received SNR using the channel quality model based on the SNR thresholds, given by [18]:

(2) $$CQI=\{\begin{array}{c} 0\;if\gamma \leq -16\\ \left|\frac{\gamma }{1.02}+16.16\right|if-16<\gamma \leq 14\\ 30\;if\;14<\gamma \end{array}$$

The channel quality estimation model adopted in this work is for the HSPDA [19] platform; however this work can be applied to any channel model. The estimated CQI value is used to characterize the state of the channel. The wireless channel is analytically modeled as a three state Markov chain with state space S = {good, fair, and bad}. To analyze the performance of the Rayleigh fading channel, the received SNR values are partitioned into three intervals of thresholds $0<\Gamma_{f}<\Gamma_{g}<\infty$ in increasing order. The channel is in a bad state if the amplitude of its SNR resides between 0 and $\Gamma_{f}$ [20] and the steady state probability is SNR CDF given by:

(3) Pb(γ)=Pr{0≤γ≤Γf}=1−exp(−Γfγ¯)

When the received SNR on the link is between $\Gamma_{f}\;$ and$\;\Gamma_{g}$, the channel is in fair state, its steady state probability is given by:

(4) Pf(γ)=Pr{Γf≤γ≤Γg}=exp(−Γfγ¯)−exp(−Γgγ¯)

In good state the received SNR on the link is above${\;\Gamma }_{g}$, the steady state probability is given by:

(5) Pg(γ)=Pr{Γg≤γ≤∞}=exp(−Γgγ¯)

Cooperative communication is employed based on quality of the direct link between the communicating node pairs.

### 4.2. Traffic Model

Individual sensor nodes generate messages for event detection in the WSN system. The arrival of messages at a node is modeled as a Poisson process. The communication is divided into time frames and each time frame has a duration period *T*. The arrival of messages is a Poisson process independent of the node state in the network at the rate $\;\lambda_{m}$ messages/s. The length of a message is geometric with mean length $\bar{m}$ packets. The arrival rate in terms of packets at each individual node will generally be given as $\lambda_{p_{i}}=\lambda_{m_{i}}\times \bar{m}$ packets per second. The number of packets arrivals $\;X_{t}$ at the *t*th frame of period T is a Poisson process of arrival rate $\lambda_{p_{i}}$ with a probability distribution given as [21,22]:

(6) $$\mathrm{Pr}\left(X_{t}=k\right)=\frac{{\left(\lambda_{p_{i}}T\right)}^{k}}{k!}exp\left(-\lambda_{p_{i}}T\right)\;k>0$$

The average number of packet arrivals for a node during a frame of period T is given by $E\left|X_{t}\right|=\lambda_{p_{i}}*T$; however depending on the roles of each node the arrival varies. The CH and RCH have high traffic load. The average packet arrival for each node i is therefore given by:

(7) $$\lambda_{{\bar{p}}_{i}}=\{\begin{array}{c} \lambda_{p_{i}},SN\\ \lambda_{p_{i}}+\sum_{k=1}^{N_{i}}\lambda_{p_{k}},CH\\ \lambda_{p_{i}}+\sum_{k=1}^{N_{i}}\lambda_{p_{k}}+\lambda_{p_{j}}+\sum_{k=1}^{N_{j}}\lambda_{p_{l}},RCH \end{array}$$

where $N_{i}\;$ and $N_{j}$ are the number of cluster members if node is CH, or number of CHs if node is an RCH. $\lambda_{p_{i}}$ is the arrival generated by node i, $\lambda_{p_{k}}$ and $\lambda_{p_{l}}$ are the arrivals by the child nodes k and j. The traffic at the RCH is the traffic for its own cluster and for the cluster it relays information for.

### 4.3. Stochastic Model for the Schedule Analysis

#### 4.3.1. Network State Analysis

Each node in the sensor network can exist in any one of the three states: sleep ($S_{1}$), active ($S_{2}$) and back-off ($S_{1})$state. The state of the network is modeled as a Markov process of state space x={0,1…K}, where x is the number of active nodes in the network during a frame. The state of the network changes from frame to frame with the capacity K as the number of schedule slots for each frame in the network. The time a node spends in the sleep state $T_{s_{1}}$is geometric with mean $1/u_{S_{1}}$, and time it spends in the back-off state $T_{s_{3}}$ is geometric with mean $1/u_{S_{3}}$. The time a node spends in the active state $T_{s_{2}}$ is defined by the mean termination rate $1/u_{S_{2}}$, indicating the duration of node in the active state. In a frame, new nodes can move to the active state, and some active nodes will terminate from the active state. Therefore the transition probability from i active nodes on one frame  t to j active nodes in the next frame  t+1 is given by:

(8) $$P_{ij}=\{\begin{array}{r} \sum_{c=0}^{i}f_{A}(j-i+c|i)*f_{T}(c|i),i<k,j>i\\ \sum_{c=i-j}^{i}f_{A}(j-i+c|i)*f_{T}(c|i),i<k,j<i\\ f_{T}(i-j|i),i-K,j<i\\ f_{A}(j-i|j),i<K,j=i\\ 0\;otherwise \end{array}$$

where $f_{A}\left(y|i\right)$ is the probability that y nodes change to the active state in the current frame given that there were i nodes in the previous frame. The probability follows a binomial distribution such that $f_{A}\left(y|i\right)=b\left(y,N-i,u_{S_{1}}\right)$. The term $\;f_{T}\left(y|i\right)$ in Equation (8) is the probability that y nodes exit the active state given that i nodes are active in the frame. This termination probability follows a binomial distribution such that $f_{T}\left(y|i\right)=b\left(y,i,u_{S_{2}}\right)$. The steady state probability $\pi_{j}$ is the probability of being in state x=j where there are j number of active nodes in a frame, this probability is derived by applying the stationary distribution to the transition matrix $\;P_{ij}$ and solving the below equations:

(9) $$\pi_{j}=\overset{K}{\underset{i=0}{\sum }}\pi_{i}P_{ij}\;,\;\overset{K}{\underset{j=0}{\sum }}\pi_{j}=1\;$$

The probability that out of the j active sensor nodes in a network of z clusters, k nodes belong to a cluster is a binomial process given by [23,24]:

(10) P(k≤CN−1|j)=∑k=0CN−1(jk)(1z)k(1−1z)j−kπj

where CN is the number of sensor nodes in a cluster.

#### 4.3.2. Node Scheduling

A sensor node is scheduled within a cluster if it has large number of packets due for transmission and low energy resources in its battery. The scheduler is cross-layer based as it involves the packets and energy level at the MAC and physical layer respectively. It is represented by a medium access decision parameter$\;\varphi_{i}$ for all contending nodes. The medium access decision parameter is defined as the ratio of the number of available packets$\;\left(n_{i}\right)$ to the remaining energy level $Er_{i}$ for each node. It is used to decide the probability of a node to win the medium. The probability that a node w, out of the k contending nodes, wins the medium for the transmission slot P(w|k) is defined by the fact that a node has the maximum value of the access decision parameter among its contending members and is defined in simulation as [25]:

(11) $$P\left(w|k\right)=\overset{k}{\underset{i=1}{\prod }}P\left(\varphi_{i}\leq \varphi_{w}\right)$$

The probability of winning the contention is the probability that a node is scheduled in a slot, that is $\;P\left(w|k\right)={\mathrm{Pr}}_{sch}$, where $\varphi_{i}$ for each individual node in a cluster is a function of the number packets arrivals and the energy level in the sensor nodes. A node that spends the most time in the active state, transmits the most packets, and has the highest utilization factor $\tau_{i}$ [26,27], hence the lowest remaining energy. Therefore the remaining energy level $Er_{i}$ is proportional to the time a node spends in the active state. The number of packets $n_{i}$ is also proportional to the arrival rate and the frame period T, giving $\varphi_{i}$ is given as:

(12) $$\varphi_{i}=\frac{\lambda_{{\bar{p}}_{i}}*T}{u_{S_{2}}\;}$$

where $\lambda_{{\bar{p}}_{i}}$ is the arrival rate of each node type defined in Equation (7). In a cluster normal nodes are scheduled based on the medium access decision parameter while CH and RCH are given priority of access as they route their packets and those of other nodes. For k normal nodes, with the same arrival statistics in a frame the probability of the nodes being scheduled is given as:

(13) Prsch=1k

where k is the total number of active nodes in a cluster.

### 4.4. Node State Analysis

**Figure 8:**
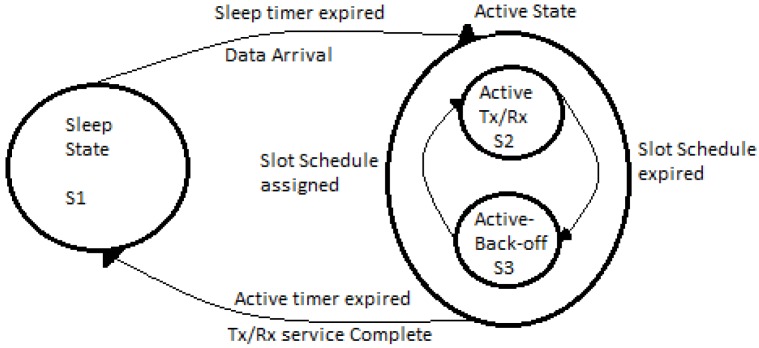
Node’s State transition Diagram.

The sensor node state behavior is modeled as a Markov process of state space S = {Sleep ($S_{1}$), Active Tx/Rx ($S_{2}$), Active back-off ($S_{3}$)} as illustrated in Figure 8. The state transition vary depending on the type of node due to their variable arrivals, however they generally follow similar behavior. A node is in the sleep state if it has no data to transmit or not receiving any data. It is in the active back-off state when it has data packets in its buffer but it is not scheduled for transmissions. When scheduled for communication it transmits its data packets in the active Tx/Rx state.

The transition probability matrix P for the sensor node state behavior is:

(14) $$P=\left(\begin{array}{ccc} P_{S_{1}S_{1}} & P_{S_{1}S_{2}} & P_{S_{1}S_{3}}\\ P_{S_{2}S_{1}} & P_{S_{2}S_{2}} & P_{S_{2}S_{3}}\\ P_{S_{3}S_{1}} & P_{S_{3}S_{2}} & P_{S_{3}S_{3}} \end{array}\right)$$

The transition probabilities are determined from the transition events.$\;P_{S_{1}S_{1}}\;$is the probability of a node continuing in the sleep state. A node will continue to stay in the sleep state when its sleep timer has not expired; it will stay in that state for a random period of time. Therefore the probability $P_{S_{1}S_{1}}$ is given by:

(15) Ps1s1=1−1uS1

A node will transit into the Active Tx/Rx state with a probability $P_{S_{1}S_{2}}$ when its sleep timer expires and it is allocated a slot schedule to transmit its data:

(16) Ps1s2=1uS1∗Prsch

where ${\mathrm{Pr}}_{sch}$ is the probability that a node is scheduled within a communication frame. A node transits from the sleep state to the back-off state with probability$\;P_{S_{1}S_{3}}$if the sleep timer expires and there is data to transmit however there are no resources for a node to be scheduled:

(17) Ps1s3=1uS1∗(1−Prsch)

A node will exit from the active Tx/Rx only if it has completed its transmission hence the probability $P_{s_{2}s_{3}}$ is defined as zero:

(18) $$P_{s_{2}s_{3}}=0$$

A node will continue to stay in the active Tx/Rx state with a probability $P_{s_{2}s_{2}}$ if it has not completed its transmission:

(19) Ps2s2=1−1uS2

If transmission of packets is complete, the node transits to the sleep state from active Tx/Rx to conserve energy; hence the probability $P_{s_{2}s_{1}}$ is given by:

(20) Ps2s1=1uS2

A node transits to the active Tx/Rx state from the active back-off state with a probability $P_{s_{3}s_{2}}$ when its back-off timer expires and it is scheduled slot, given by the following:

(21) Ps3s2=Prsch∗1uS3

A node transits to the sleep state only when its buffer is empty. Therefore the node cannot transit from the back-off state to sleep state since in the back-off state it has not serviced all its packets, giving the probability $P_{s_{3}s_{1}}$as zero:

(22) $$\;P_{s_{3}s_{1}}=0$$

A node stays in the active back off state with a probability $P_{s_{3}s_{3}}$ if its back-off timer has not expired or if its back-off timer expires and it is not scheduled for transmission, given by:

(23) Ps3s3=(1−1uS3)+(1uS3∗(1−Prsch))=1−Prsch∗1uS3

The mean termination rate from the active state $u_{S_{2}}$ is determined from the average time a node spends in the active state. The time a node takes in the active state is a function of the available packets n and the time to transmit a single packet. Generally the number of packets for a node will be given as:

(24) $$n=\overset{3}{\underset{i=1}{\sum }}n_{s_{i}}$$

where $n_{s_{i}}=\frac{\lambda_{{\bar{p}}_{i}}}{u_{s_{i}}}$, indicates the arrivals during each of the three node states. Based on the fact that the scheduler alternates the states of the nodes between active and back-off, the duration of time in these states will depend on the probability of the node being scheduled. Therefore the average time a node spends in active state ${\bar{T}}_{S_{2}}$ is given by:

(25) T¯S2= λp¯i Tts∗(1us1+Prschus2+1−Prschus3 )

where $T_{ts}$ is the slot time to transmit a single packet. The steady state probabilities $\;\rho_{s_{1}}$, $\rho_{s_{2}}$ and $\;\rho_{s_{3}}$ are obtained through solving for the equations below:

(26) $$\rho_{s_{j}}=\overset{3}{\underset{i=1}{\sum }}\rho_{s_{i}}P_{s_{i}s_{j}}\;,\;\overset{3}{\underset{j=1}{\sum }}\rho_{s_{j}}=1$$

### 4.5. Performance Measures

#### 4.5.1. Energy Consumption Model

The energy consumed by radio communication at any time by a node is the function of the amount of energy it dissipates in each state, generally given as:

(27) $$E_{n}=\overset{3}{\underset{i=1}{\sum }}E_{s_{i}}$$

where $E_{s_{i}}$ is the amount of energy the sensor node dissipates in a state. The energy used in the sleep state is given by:

(28) $$E_{s_{1}}=Pw_{s_{1}}*T_{s_{1}}$$

where $Pw_{s_{1}}$ is the unit time energy spend in the sleep state and $T_{s_{1}}$ is the mean time the node takes in the sleep state. The energy dissipated by a node in the back-off state is given by:

(29) $$E_{s_{3}}=Pw_{s_{3}}*T_{s_{3}}$$

where $Pw_{s_{3}}$ is the unit time energy spend in the back-off state and $T_{s_{3}}$ is the mean time a node spends in back-off state. The energy $E_{s_{2}}$ in the active communication state comprises of the transmitting and the receiving energy and the time it takes to service the packet at the node depending on the length of the packet:

(30) Es2=(Pwtx+Pwrx)∗ (Ts2¯∗LpacbitsRbits/sec+LackbitsRbits/sec + LrtsbitsRbits/sec+LschbitsRbits/sec+LrsubitsRbits/sec+LctsbitsRbits/sec)

where $Pw_{tx}$ and $Pw_{rx}$ are the unit energy to transmit and receive a bit of information respectively and $\bar{T_{s_{2}}}$ is the average time a node is active. The energy for each cluster is given as the sum of energy used for transmission of every cluster member, the total energy utilized in the cluster is given by:

(31) $$E_{tot-intra}=\overset{CN}{\underset{n=1}{\sum }}E_{n}$$

The energy consumed in inter-cluster communication depends on the communication of the cluster head nodes. The communication is either direct or cooperative; the energy consumed by each type of node depends on the amount of time it spends in each of the states based on individual role in the network. This also depends on the traffic of each node as detailed in Equation (7).

#### 4.5.2. Lifetime

The estimated lifetime of the node is determined from its initial energy and the total energy it uses for transmission [28,29]:

(32) $$E\left|L\right|=\frac{E_{0}-E_{u}}{E_{tot}}$$

where $E_{u}$ is the energy level at which the sensor is considered unusable, which is decided on implementation, $E_{0}$ is the node’s initial deployment energy level and $E_{tot}$ is the sum of the energy for intra-cluster and inter-cluster communication.

### 4.6. Delay

The delay performance result in this work is based on a simulation model. The average end to end delay is considered as the time from packet inception to successful receipt at the destination. With the effect of fading the delay is dependent on the quality of the channel as it impacts the number of retransmissions. For direct the average delay is given by:

(33) $$D_{Avrg_{direct}}=T_{rts}+T_{cts}+T_{dat}+T_{ack}$$

where $T_{rts}$ is time duration to send RTS by source node $T_{cts}$ is time to send CTS by destination node, $T_{dat}$ is time duration for data transmission for a single packet, and $T_{ack}$ is the delay to send the acknowledgement packet.The transmit delay of the each packet is the packet length in bits over the average data transmitrate of the network given by:

(34) Tpactype=LpactypebitsRbits/s

where $T_{pac_{type}}$ is the delay per packet type, $L_{pa{c_{type}}_{bits}}$ is the length of the packets in bits and $R_{bits/s}$ is the data rate. The delay incurred for cooperative transmission of packets between source, relay and destination is given by:

(35) $$D_{Avrg_{coop}}=T_{rts}+T_{cts}+T_{hcts}*RL+T_{rsu}+T_{dat_{sr}}+T_{dat_{rd}}+T_{ack}$$

where RL is the number of possible relay nodes, $T_{rsu}$ is the amount of time incurred to transmit the relay selection update and $T_{dat_{sr}}$ and $T_{dat_{rd}}$ is the time to transmit the data from source to relay and from relay to destination in a cooperative transmission.

## 5. Performance Results

This section presents the simulation and analytical results for the proposed energy-efficient MAC protocol for cluster-based wireless sensor networks (EEMACCSN) MAC framework. The network is simulated for 100 immobile sensor nodes randomly distributed over a 2-dimensional geographical network area of 500 m × 500 m. Each node has two properties, namely their location coordinates and the energy level used to determine their cluster membership. The nodes with the highest energy level are determined and then clusters are formed using the Voronoi algorithm. The inter-arrival of packets on each node in the network is exponentially distributed, following a Poisson process and independent per sensor node. The simulation results are obtained from an event driven custom built Visual C++ simulator. The simulation parameters are shown in Table 2 similar to the parameters used in [10]. Some parameters are varied in the simulations to depict different scenarios as indicated on the results.

**Table 2:** Simulation parameters.

Number of Nodes	100
Area	500 m × 500 m
Packets arrival rate	Varied from 0.01 to 0.1 packets per second
Data Packet	250 bytes
Length Packets (RTS,CTS,ACK)	18 bytes
Length Packets (RSU,SCH)	16 bytes
Initial Energy Eo	randomly distributed between (0–50) Joules
Pw_transmit	462 mW
Pw_receive	346 mW
Pw_idle	310 mW
Pw_sleepSNR threshold at receiver	100 μW9 dB

**Figure 9:**
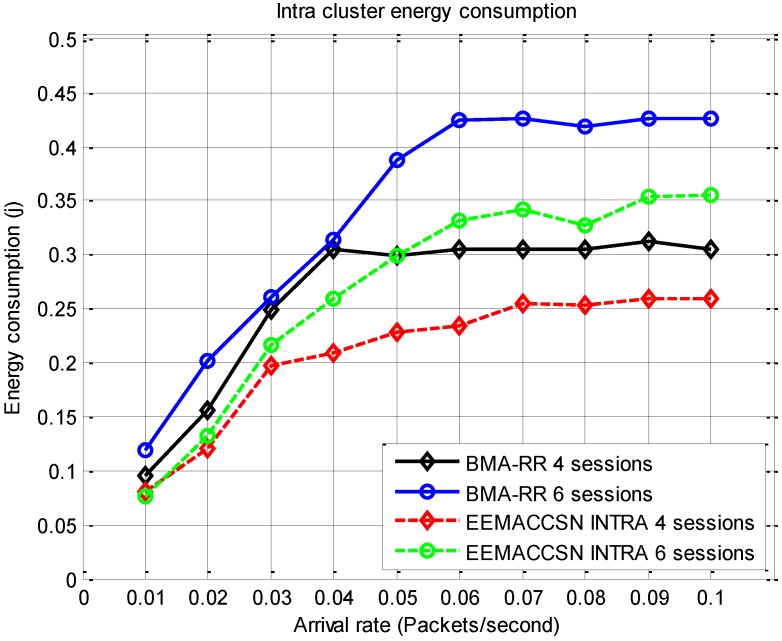
Intra-cluster energy consumption per node *vs.* arrival rate.

The performance of the proposed EEMACCSN protocol is first compared to the BMA-RR [10], a MAC protocol for cluster based sensor networks. Figure 9 presents the results of energy consumption per node during intra-cluster communication round for variable arrival rates. The proposed MAC framework intra-cluster communication is compared with BMA-RR [10] energy consumption. The intra-cluster communication involves the communication of the normal cluster member nodes with their cluster head node, within a cluster. The energy consumption in the network increases with the increase in the arrival rate, this is due to the fact that at high arrival rates the network spends most of its time in the transmitting the packets which contributes to the high energy consumption. It is also observed that the energy consumption is high when the number of sessions is increased as more communication slots are assigned to nodes in a round. The energy consumption at arrival rates λ> 0.04 also reach a steady point due to the fact that the slots in each session are fixed, and only maximum available slots can be allocated just as in [10]. From the results we can deduce that EEMACCSN protocol improves the network energy consumption. This is due to the following; firstly the adaptive energy efficient cross-layer schedule for the medium access utilized by EEMACCSN. Secondly idle time is reduced during the sensor nodes contention period as only nodes that have data contend in EEMACCSN, unlike BMA-RR where all nodes are active during the contention period. Lastly the clustering algorithm of EEMACCSN allows nodes to defer transmission of announcements if a message from a higher energy node is heard leading to energy saving on such nodes.

**Figure 10:**
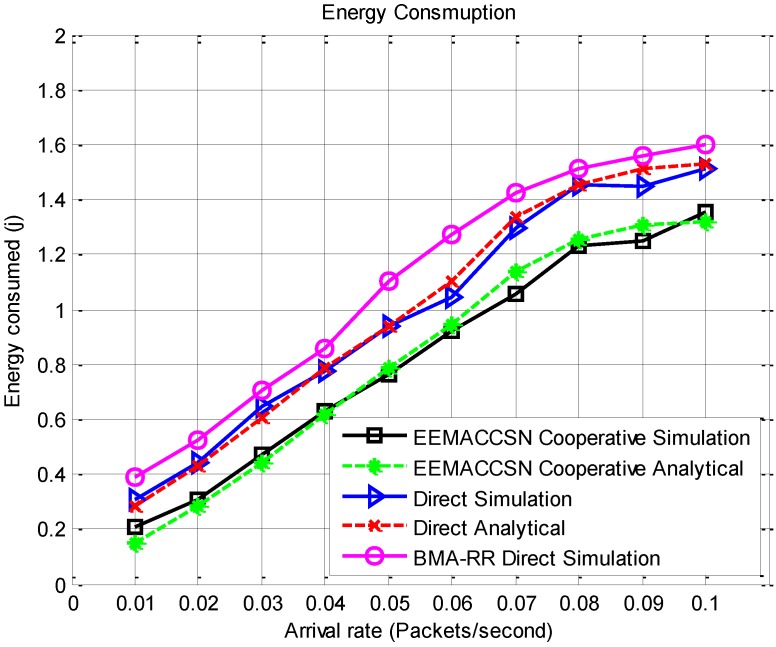
Total energy consumption per node *vs.* arrival rate.

Figure 10 presents the performance of the proposed EEMACCSN protocol in direct and cooperative communication inter-cluster communication and the modified BMA-RR with direct inter-cluster communication. The results are for the complete protocol communication including communication within a cluster and between clusters and the base station. As observed, the energy consumption in the network increases with an increase in the arrival rate. The lowest energy consumer is the cooperative EEMACCSN, then direct EEMACCSN and lastly the modified BMA-RR for direct communication. EEMACCSN protocol improves the network energy consumption. This is due to the improved reliability by cooperative communication which reduces the probability of error occurrence in transmission reducing the need for packet retransmissions. The reduced number of retransmission means node spends little time in the active state leading to energy conservation. The analytical models are closely verified by the results as both the analytical and the simulation results are in agreement.

**Figure 11:**
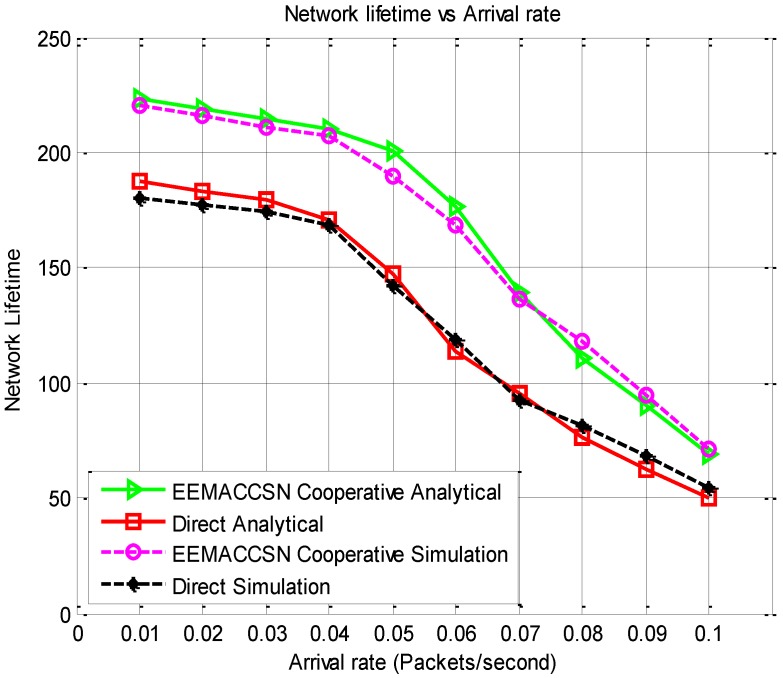
Network lifetime *vs.* arrival rate.

Figure 11 presents the network lifetime results of the proposed framework. The network lifetime depends on the energy consumption of the individual nodes as their lifetime determines the connectivity of the network. The higher the energy consumption on the sensor nodes the faster the nodes energy gets depleted, this has a direct impact on the lifetime of the sensor network. It is observed that the lifetime decreases with the increase in the arrival rate due to the fact that the nodes radio spend most time in the communication mode which consumes more energy on the sensor nodes. The lifetime of the developed EEMACCSN protocol with cooperative communication is longer than when direct communication is used. This is attributed to the high energy consumption in the direct communication, due to the high probability of retransmission occurrence since there is no measure to mitigate the fading effects of the wireless channels in direct transmission.

**Figure 12:**
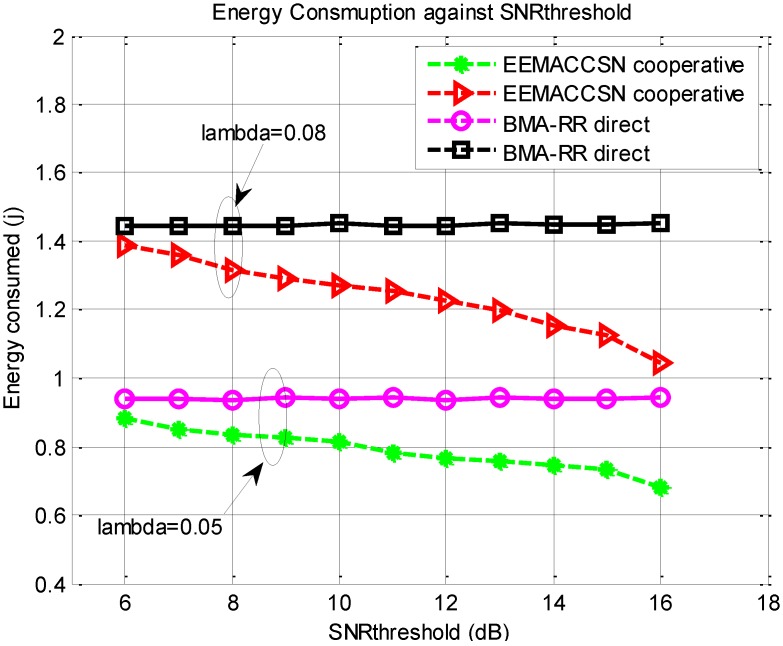
Energy consumption *vs.* SNR_threshold.

Figure 12 presents the energy performance of the proposed MAC protocol over variable SNR threshold with varying arrival rate settings. 

For cooperative communication the energy consumption in the network decreases with the increase in the SNR threshold. The high SNR threshold means that the cooperative communication will often be employed in the network thereby improving the energy performance through the use of more reliable high capacity channels. However, for cooperative communication, the SNR thresholds in the system should be selected with great care, as they impact the way cooperative systems behave. Low thresholds result in unreliable channels as direct transmission is allowed, even when the received SNR is low, at the same time very high thresholds lead to delay and waste of energy, where additional nodes are used to cooperate while a single transmission would provide enough signal quality. For direct communication, as expected, the energy consumption is not affected by the SNR threshold, since the system does not check the threshold to decide on the communication. It is observed from the results the increase in the traffic load influences more energy consumption in the network. The proposed EEMACCSN protocol provides better energy performance with increasing SNR threshold than BMA-RR with direct transmission.

**Figure 13:**
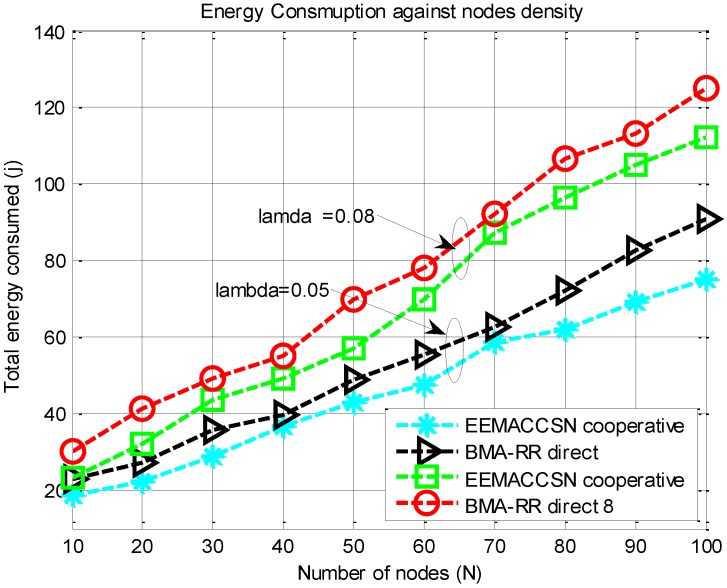
Energy consumption against number of nodes.

Figure 13 presents results for total energy consumption against increasing network node density for variable data arrival rates. As the network size increases, the interference and overhead for communication packets increases. The number of control packets transmitted to avoid collisions increases, leading to more energy consumption for transmission of communication packets. Increasing the number of network nodes also increases the likelihood of interference during packet transmissions, which reduces the SNR of transmitted packets, leading to more possible retransmissions for reliable communication. Also, since the arrival is independent on each sensor node, increasing the node density in the network increases the arrival in the network leading to increased total energy consumption. Figure 13 illustrates that the EEMACCSN provide minimal energy consumption than the BMA-RR in response to node density, due to its better adaptive coordination of the medium access and its ability to improve the signal strength of the received packets at the destination through cooperation.

**Figure 14:**
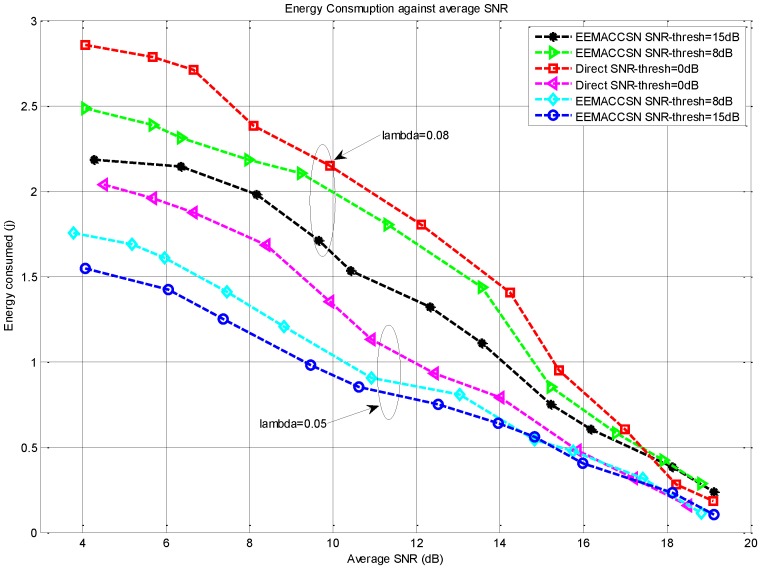
Energy consumption per node against average SNR.

The results in Figure 14 depict the behavior of the EEMACCSN against average received SNR in the network, for variable SNR thresholds. It indicates that the energy consumption reduces with the increase of the received SNR values, since the number of retransmission is reduced due to high channel quality. It is observed at low average SNR, the energy consumption of the high SNR threshold model is better, as it compensates the capacity of the channel by diversity, while others are prone to retransmission due to SNR values, resulting in more energy consumption due to retransmissions. At high average SNR values, the energy performance of all the models is the same in terms of energy, as they all fall back to the direct transmission model, and the energy consumption is lower due to the high capacity channel. Though cooperative channels are considered to be prone to signalling packets overhead, however their effect is minimal as cooperation is implemented only when direct communication fails at low SNR. At higher SNR cooperative transmission falls back to direct transmission cutting off the signalling overhead. Also the overhead of a small size signalling packet is smaller than the one caused by the retransmission of a communication packet on the direct low quality link.

**Figure 15:**
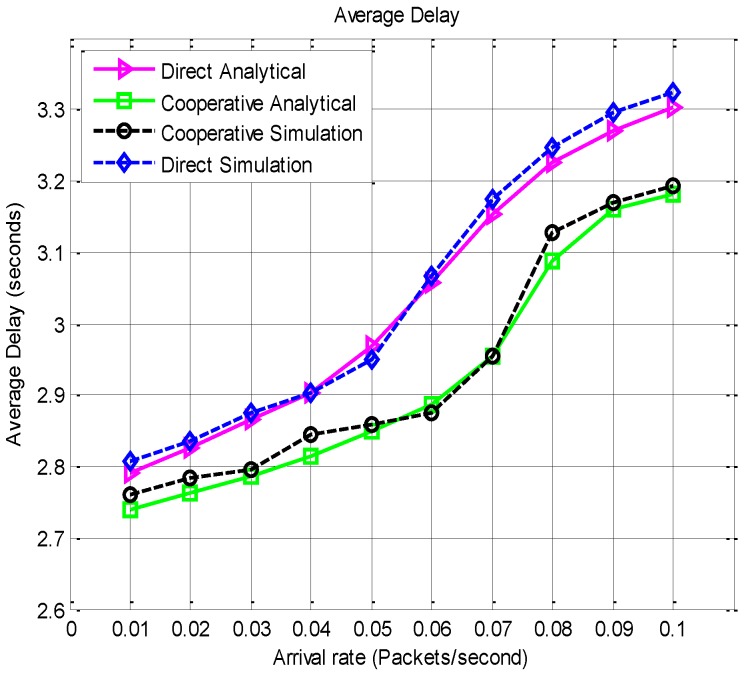
Average end to end delay *vs.* arrival rate.

The average end to end delay performance results are presented in Figure 15. The end to end delay is averaged as the time from inception to the correct reception at the destination, including the time for retransmission.

It is observed from Figure 15 that the delay performance is better with the proposed MAC under cooperative communication. Though cooperative transmission adds another node and an extra slot in the transmission, it does however come with the benefit of reduced retransmission due to diversity and enhanced channel quality.

**Figure 16:**
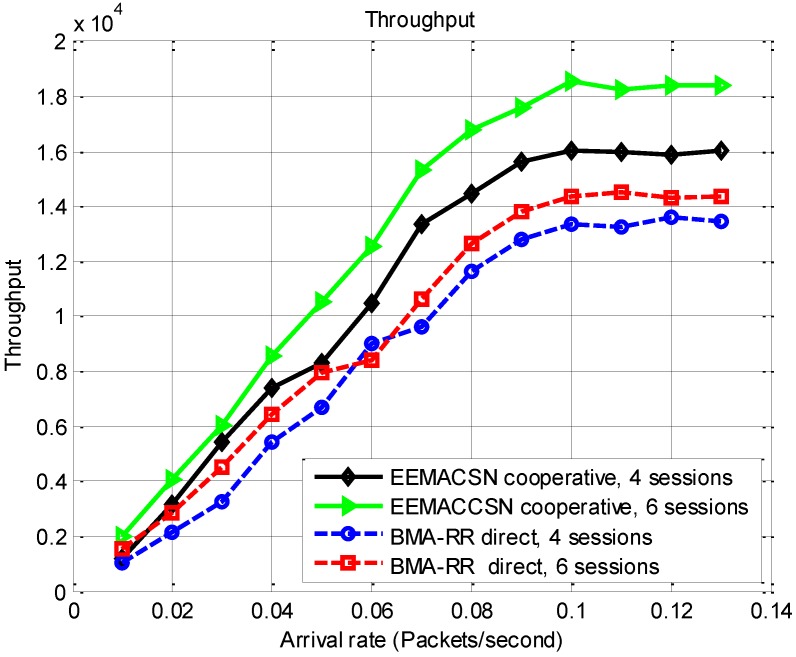
Throughput *vs.* arrival rate, SNR threshold 9 dB.

In Figure 16 the throughput results are presented for EEMACCSN framework and the BMA-RR protocol with direct transmission for different number of communication sessions. It is observed form the results in this figure that a higher throughput is achievable if the number of sessions is high. This is due to the fact that the system spends most of its time in the transmit state, than when there are few communication session. It is also observed that with the use of cooperating channels the number of successful transmission increases. The results also show a steady throughput as the arrival rate in the system increases, indicating that throughput is also dependent on the systems available resources.

**Figure 17:**
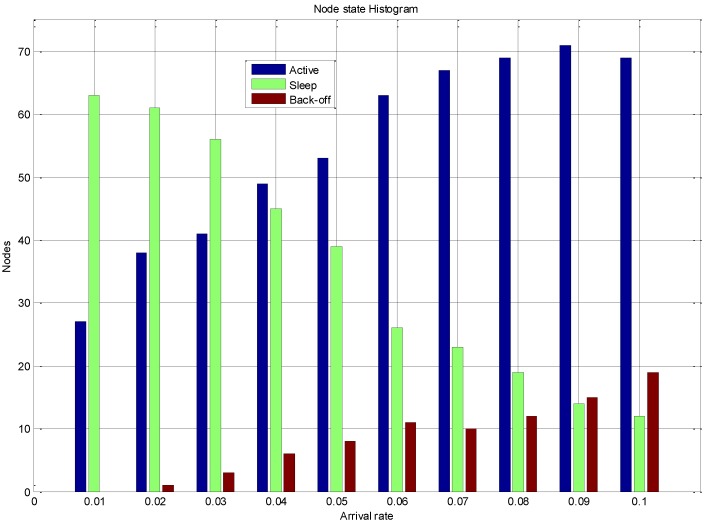
EEMACCSN node state behaviour *vs.* arrival rate.

Figure 17 displays the dynamics of the system in terms of the nodes state for variable traffic rates. At high arrival rates, the number of nodes in the active state increases and in the back off state it also increases. This is due to fact that at high traffic rate most nodes have data to send, but only the nodes that have gained access to the channel transmit their packets. Also at low data rate it is observed that there are a large number of nodes in the sleep state. The nodes that do not have data to transmit are scheduled for the sleep state to conserve the energy during the communication frame. The traffic on the sensor node has an impact on the state behaviour of the sensor node, the nodes experiencing low data arrival rates have the higher probability of staying in the sleep and hence conserving energy resources. However the number of back-off state increases with the increase of the data traffic as more nodes contend for communication resources. The system dynamics further validate the energy consumption, lifetime and throughput results presented.

**Figure 18:**
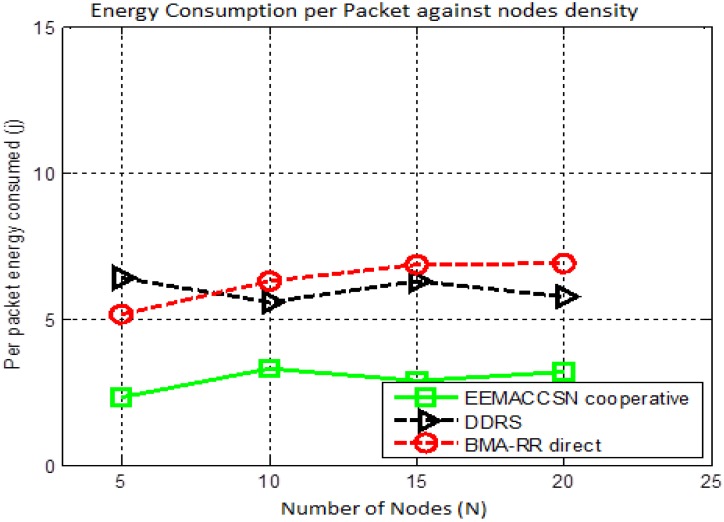
Energy performance *vs.* number of nodes (N).

Figure 18 illustrates the system energy performance against the varying node densities in the network. The proposed system model performance is compared with a DDRS cooperative MAC [30]. DDRS is a reactive cooperative MAC protocol employing cooperative transmission in the event that Direct Transmission Mode has failed. EEMACCSN and DDRS are both cooperative MACs, with EEMACCSN having a better energy performance. The results in Figure 18 further demonstrate that a good MAC protocol design has great influences on the total energy consumption of the networks.

## 6. Conclusions

This work proposes a dynamic MAC framework for cluster-based wireless sensor networks. The proposed model features an adaptive cross-layer scheduling for the node activity. The sensor nodes transceiver module in the proposed model transitions between three states—sleep, active and back off—depending on the schedule. It also features a cooperative communication between clusters and the base station. The cooperation of nodes is decided based on the received SNR—if it is below the threshold, the nodes cooperate to enhance the reliability of the network. An analytical model for the proposed framework was developed and its performance evaluated based on the results. The results indeed show the improved performance on the energy consumption, channel capacity, throughput and delay on wireless sensor by using the proposed framework. The energy performance has a direct positive impact on the lifetime of the wireless sensor network as indicated by the results and this improvement of the network lifetime overcomes the main design challenge of this network type. The accuracy of our model is concluded from the consistency observed from the analytical and simulation results.
